# Suicide Risk, Alcohol Consumption and Attitudes towards Psychological Help-Seeking among Lithuanian General Population Men, Conscripts and Regular Active Duty Soldiers

**DOI:** 10.3390/ijerph20043457

**Published:** 2023-02-16

**Authors:** Egle Mazulyte-Rasytine, Dovile Grigiene, Danute Gailiene

**Affiliations:** Suicide Research Centre, Institute of Psychology, Faculty of Philosophy, Vilnius University, 01513 Vilnius, Lithuania

**Keywords:** military, suicide, suicide risk, alcohol, help-seeking, professional psychological help, attitudes

## Abstract

The aim of this study was to investigate the relationship between suicide risk, alcohol consumption, and attitudes towards professional psychological help among Lithuanian general population men, conscripts, and regular active duty (AD) soldiers. In total, 1195 Lithuanian adult males participated in the study: 445 men from the general population, 490 conscripts, and 260 regular AD soldiers from the Lithuanian Armed Forces. The study’s measures included: general suicide risk, alcohol consumption levels, frequency of using alcohol as a means to suppress difficult thoughts and feelings, and attitudes toward psychological help. Both military samples showed significantly lower suicide risk than men from the general population. Alcohol use as a means to suppress difficult thoughts and feelings was the most significant predictor of suicide risk and a significant mediator between alcohol consumption and suicide risk in all study groups. Another significant suicide risk predictor and mediator between alcohol consumption and suicide risk—i.e., the value of seeking psychological treatment—was found only in the conscript sample. Results of the current study suggest that there is an opportunity for intervention aimed at the attitudes toward seeking professional psychological help for conscripts. However, that might not be the case for regular AD soldiers, nor the general population of Lithuanian men.

## 1. Introduction

Historically, military suicides have been significantly less common than civilian suicides in the general population, but the trend has recently been changing, for example, with the number of military suicides significantly increasing in the United States (US). Suicide rates among active duty military personnel in the US have been gradually increasing, peaking in 2020 [1]. A study by Jones and colleagues [2] indicated an increase in the prevalence of self-harm (including suicide attempts) among the United Kingdom (UK) serving military personnel, although the increasing numbers do not correspond to the actual changes in the suicide rates observed over the same period of time. Willmund and colleagues [3] also reported an upward tendency in suicide rates in the German Armed Forces with military suicide rates surpassing the general German population rates in 2014 and 2015.

After several decades of decline in the number of suicides, Lithuania remains among the leading countries in Europe in terms of the suicide rate in the general population [4]. Yet, the overall suicide rates among Lithuanian active duty military personnel remain significantly lower [5]. Due to the unique context (the suicide rates in Lithuania being extremely high in the general population), it is not clear whether the same suicide risk factors would be relevant, as the vast majority of studies on suicidal behaviour in the military have been carried out in countries with relatively low overall suicide rates.

The World Health Organization indicates the harmful use of alcohol as one of the most important suicide risk factors; about one-fifth of suicides can be attributed to alcohol consumption [4]. Research studies support a significant association between alcohol consumption and suicidal behaviour in both the general [6,7] and military populations [8]. Moreover, the problem of harmful alcohol consumption might be particularly pronounced in military populations. For example, excessive alcohol consumption among soldiers in the UK is significantly higher than in the general population [9]. Jones & Fear [10] argue that it is possible that certain characteristics which ensure the soldiers’ success on the battlefield put them at risk for alcohol abuse, i.e., soldiers need to be risk-taking individuals, ready to take risks of death or injury.

Although there’s a lack of research regarding alcohol use within the Lithuanian Armed Forces, in general, Lithuania, as a country is characterised by particularly high alcohol consumption, placing itself among the leading countries in Europe in terms of the amount of alcohol consumed [11]. Recent suicidological studies conducted in Lithuania also show that harmful alcohol consumption is associated with a higher suicide risk [12].

The stigma associated with experiencing mental health problems remains one of the most important factors influencing the difficulties in treating mental health disorders [13]. A systematic review performed by Hom and colleagues [14] showed that psychological help is underutilised in the military population, and only less than a third of soldiers experiencing mental health difficulties seek help. The most common barriers to seeking help are: the impact on a military career, stigma, structural/logistical barriers (availability), mistrust of help providers, and negative attitudes towards treatment. As for the general population, people prefer to seek help from lay sources, and many individuals, particularly males, choose not to seek any form of help for mental health problems [15]. Men are less prone than women to display positive help-seeking attitudes, particularly related to common mental health issues [16] and studies highlight a trend of delayed help-seeking among men when they become ill [17]. Additionally, alcohol use is associated with a lower likelihood of seeking help [18].

The aim of the current study was to investigate the relationship between suicide risk, alcohol consumption, and attitudes towards professional psychological help within the three study groups: Lithuanian general population men, conscripts, and regular active-duty soldiers. The conscript group was analysed separately from the regular active duty soldiers, due to the different nature of their military service. In Lithuania, all young male citizens can be conscripted into the Lithuanian Armed Forces for a 9-month mandatory military service. Due to the very uneven gender ratio in military groups (only around 12% of soldiers are female) compared to the general population and significant gender patterns in suicidal behaviour (in Lithuania the ratio of male to female suicide rates is approximately 6:1 [4]), alcohol consumption (in Lithuania men consume up to four times more alcohol than women [11]), and help-seeking (men are less likely to seek help for mental health difficulties [15]), only male samples were chosen for the purpose of the current study.

## 2. Materials and Methods

### 2.1. Participants

In total, 1195 Lithuanian adult males from three different samples—men in the general population, conscripts, and regular active duty (AD) soldiers—participated in the current study. The detailed sociodemographic characteristics of all three samples are presented in Appendix A (see Table A1).

The general population sample consisted of 445 adult men (age between 18–59, *M* = 26.60, *SD* = 12.42) from the Lithuanian general population quota (based on age and residential area) sample. The original collected general population sample consisted of 562 men (a detailed description of the sample is published elsewhere [19]); however, for the purpose of the current study, the oldest age groups of 60+ were dropped to match the overall age interval with that of the regular active duty soldier sample.

The conscript sample consisted of 490 young males (age between 18–29, *M* = 20.85, *SD* = 1.48) enlisted in the 9-month mandatory military service. The average duration of their service at the time of the survey was 6.1 months (*SD* = 2.37). Compulsory military service has been re-introduced in Lithuania in 2015 and the obligation is applied to male Lithuanian citizens aged 18 to 23. Conscription is on a lottery basis. There is also a possibility to participate in this service on a voluntary basis—both male and female citizens of Lithuania of age 18-38 years have the possibility to enlist for the service on their own accord. However, the enlistment method (volunteered vs. drafted) was not documented in the current study. Only the male subsample was used for the purpose of the study.

The regular AD soldier sample consisted of 260 soldiers (age between 19–58, *M* = 31.87, *SD* = 8.95) from the Lithuanian Armed Forces quota (based on military rank) sample. Only the male subsample was used for the purpose of the current study. The average duration of regular AD soldiers’ military service at the time of the survey was 9.91 years (*SD* = 8.19).

### 2.2. Procedure

The data collection of both military samples (conscripts and regular AD soldiers) was carried out in the paper-and-pencil format between February 2022 and July 2022. All soldiers were asked to participate in the survey on a voluntary basis. Soldiers were recruited face-to-face in their units. The interviewers were military psychologists instructed by the first author of this article. The survey questionnaire was anonymous; no personal information was gathered, and confidentiality was ensured. Soldiers were also provided with an empty sealable envelope to put their filled-in questionnaires to ensure extra confidentiality if needed. As the questionnaire included sensitive questions about suicidal behaviour and other psychological difficulties participants might be experiencing, all participants were provided with information about emotional support and professional psychological help options within and outside the military.

The data of the general population sample came from another research study carried out recently by the Suicide Research Centre between June 2020 and April 2021. A detailed description of the general population data collection procedure is published elsewhere [19].

### 2.3. Instruments

The Suicide Behaviors Questionnaire–Revised (SBQ-R) is a brief instrument aimed to assess persons’ suicidality [20]. SBQ-R consists of four items from the original Suicidal Behaviours Questionnaire (SBQ) that assess the life-long history of suicidal ideation and attempts, frequency of past-year suicidal ideation, threats of suicide, and the likelihood of suicide completion. The total score on the measure ranges from 3 to 18, with higher scores reflecting a greater risk for suicide. The Lithuanian version of the SBQ-R demonstrates good psychometric properties [19]. A preliminary cut-off score of seven or higher is suggested for the adult non-clinical sample [20]; however, the cut-off score is not validated in the Lithuanian population, therefore should be used with caution. The internal consistency of SBQ-R in the current study sample was good (*alpha* = 0.81)

The Alcohol Use Disorders Identification Test–Consumption Questions (AUDIT-C) is a brief screening instrument that reliably identifies heavy drinking [21]. The AUDIT-C consists of the three alcohol consumption questions from the full Alcohol Use Disorders Identification Test (AUDIT). It is scored on a scale of 0–12 (each AUDIT-C question has five answer choices valued from 0 points to 4 points). In men, a total score of four or more suggests hazardous drinking or active alcohol use disorders, the same cut-off score is suggested to be used also in the Lithuanian population [22]. Generally, the higher the score, the more likely it is that a person’s drinking is affecting his or her safety. The internal consistency of AUDIT-C in the current study sample was good (*alpha* = 0.80).

Additionally, study participants were asked to rate how often they drink alcohol as a means to suppress difficult thoughts and feelings (SUPPRESS) on a scale of 0–4 (‘never’ to ‘very often’).

The Attitudes Toward Seeking Professional Psychological Help–Short Form scale (ATSPPH-SF) is a 10-item questionnaire to assess persons’ professional help-seeking attitudes [23]. There are various factor structures for the ASPPH-SF proposed in the literature [24,25]; a two-factor solution suggested by Elhai and colleagues [24] was chosen for the current study as its model fit in the Lithuanian general population sample was shown to be the best [12]. Each of the two subscales—Openness to Seeking Treatment for Emotional Problems (OPENNESS) and Value and Need in Seeking Treatment (VALUE)—consist of five items and in total are scored on a scale of 0–15 with a higher score indicating more positive attitudes towards seeking professional help. The internal consistency of ATSPPH-SF in the current study sample was good (*alpha* = 0.83) for the OPENNESS subscale and acceptable (*alpha* = 0.74) for the VALUE subscale.

### 2.4. Data Analysis

Statistical data analysis was carried out using the software environment for statistical computing and graphics R [26] along with RStudio [27], an integrated development environment for R.

Descriptive statistics and box plots of all study variables are provided in the Supplementary Materials (see Table S1 and Figure S1). No deviations from normality in terms of variable skewness and kurtosis were observed, apart from the variables of suicide risk (SBQ-R) and alcohol use as a means to suppress difficult thoughts and feelings (SUPPRESS). The data on these variables were slightly skewed (1.88 for SBQ-R and 1.72 for SUPPRESS) and the kurtosis was slightly higher (3.86 for SBQ-R and 2.66 for SUPPRESS) than would be expected in a normally distributed measure. There were some outliers in the data on SBQ-R and SUPPRESS; however, it was decided not to remove them, because they are legitimate observations that are a natural part of the population due to the rarity of high suicide risk and high deliberate alcohol use to relieve psychological stressors. Given that these measures were used in a general population sample, some deviation from the normal distribution could be expected; therefore, because the deviation was small and the sample size was large, no impact on the accuracy of the statistical test was expected and no transformation was used.

The between-group (i.e., study sample) comparison analyses were carried out using ANCOVAs with age as a covariate. All other analyses within each study sample were carried out without adjusting for age. Associations between the main study variables were assessed using the Pearson correlation coefficient separately in each sample. Separate multiple regression analyses in each study sample were conducted to evaluate the prognostic effects of alcohol consumption, alcohol use as a means to suppress difficult thoughts and feelings, openness to seeking psychological treatment, and value in seeking treatment (independent variables) on suicide risk (dependent variable). The mediation analyses were conducted using the PROCESS 4.1v macro for R created by Hayes [28]. For the general population sample and regular AD sample simple mediation models with one mediator were estimated. For the conscript sample, a serial multiple mediator model was chosen due to the significant correlation between the mediators [28].

## 3. Results

### 3.1. Comparison of Suicide Risk, Alcohol Consumption, and Attitudes towards Psychological Help between General Population Men, Regular AD Soldiers, and Conscripts

The comparison of the overall suicide risk, alcohol consumption levels, frequency of alcohol use to suppress difficult thoughts and feelings, attitudes about psychological help value, and openness to psychological help between general population men, conscripts, and regular AD soldiers are presented in Table 1.

Study groups differed significantly in their suicide risk, with general population men reporting statistically significantly greater suicide risk compared to both military samples. The comparison of the proportions of study participants who might be considered at high risk for suicide showed that all three study samples differed from each other statistically significantly, χ^2^ (2, *N* = 1167) = 61.26, *p* < 0.001. The largest percentage (31.15%) of men at high risk for suicide was found in the general population sample, a significantly lower one (21.01%) among conscripts, and the lowest one (5.65%) in the regular AD soldier sample. On the contrary, there were no statistically significant differences in alcohol consumption between the study groups. Although there was a slightly lower percentage (43.55%) of risk drinkers among regular AD soldiers than among general population men (52.60%) and conscripts (50.10%), the difference was not statistically significant, χ^2^ (2, *N* = 1172) = 5.27, *p* = 0.072. However, the proportion of abstainers between study groups differed statistically significantly, χ^2^ (2, *N* = 1172) = 15.20, *p* < 0.001. The largest proportion of abstainers was found among conscripts (24.95%), a lower one—among regular AD soldiers (18.15%), and the lowest—among general population men (14.90%). Yet, the statistically significant difference was found only between a conscript and general population men groups. Furthermore, general population men drank alcohol to suppress their difficult thoughts and feelings statistically significantly more often than regular AD soldiers. Both groups did not differ statistically significantly from the conscripts. However, conscripts reported perceiving the value of professional psychological help as significantly lower than both general population men and regular AD soldiers, as well as being significantly less open for such help.

### 3.2. Relationship between Suicide Risk, Alcohol Consumption and Attitudes towards Psychological Help

Correlations among all study variables were calculated in each study group separately and are provided in Appendix B (see Table A2, Table A3 and Table A4). There were statistically significant positive links between suicide risk and alcohol consumption as well as alcohol use as a means to suppress difficult thoughts and feelings among all study groups. The relationship between participant’s attitudes towards professional psychological help and suicide risk varied in different samples; both the perceived value of psychological help and openness to psychological help were significantly positively correlated with suicide risk in general population men; however, in the conscript sample the perceived value of psychological help was significantly negatively correlated with suicide risk and no statistically significant relationship between both attitudinal variables and suicide risk was found in the regular AD soldier sample.

In order to assess the prognostic power of alcohol consumption and attitudes towards professional psychological help-seeking to suicide risk, three separate multiple regression analyses were conducted in each of the study samples (Table 2). It turned out that alcohol use as a means to suppress difficult thoughts and feelings was the only stable statistically significant predictor variable of suicide risk in all three study samples. However, the value of psychological help was also a statistically significant suicide risk predictor in the conscript sample with a lower perceived value of professional psychological help predicting higher suicide risk.

**Figure 1:**
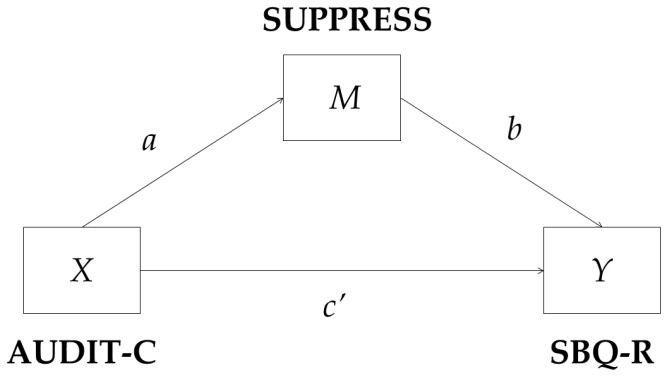
A statistical diagram of the mediation model tested for both general population men and regular AD soldier samples data.

Alcohol use as a means to suppress difficult thoughts and feelings overruled the statistically significant relationship between alcohol consumption and suicide risk in all regression models; therefore, a mediation model with alcohol use as a means to suppress difficult thoughts and feelings mediating the relationship between alcohol consumption and suicide risk was tested in the general population men and regular AD soldier samples (see Figure 1). Regression coefficients, standard errors, and model summary information are presented in Table 3. The results confirmed that alcohol use as a means to suppress difficult thoughts and feelings was a statistically significant mediator of the relationship between alcohol consumption and suicidality (Indirect effect Bootstrap 95% CI [0.055, 0.174] in the general population men sample and [0.012, 0.145] in the regular AD soldier sample). The indirect effect was positive meaning that a link between higher alcohol consumption and higher suicide risk is explained by the higher frequency of alcohol use as a means to suppress difficult thoughts and feelings in both the general population men and regular AD soldier samples.

**Figure 2:**
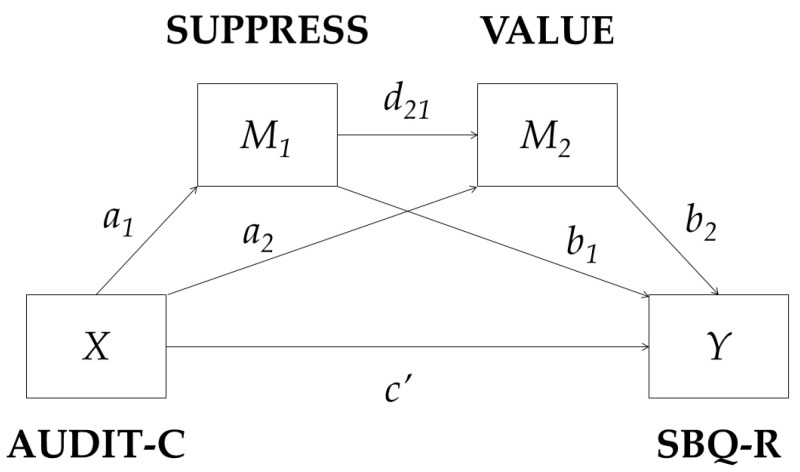
A statistical diagram of the serial multiple mediator model tested for the conscript sample data.

As the value of psychological help remained a statistically significant suicide risk predictor together with alcohol use as a means to suppress difficult thoughts and feelings in the conscript sample and both variables were correlated significantly, a serial multiple mediator model was tested (see Figure 2). Regression coefficients, standard errors, and model summary information are presented in Table 4. Identically to the previously tested mediation models in general population men and regular AD soldier samples, the indirect effect of alcohol consumption on suicide risk through alcohol use as a means to suppress difficult thoughts and feelings was also significant (Bootstrap 95% CI [0.035, 0.140]) in the conscript sample. The second indirect effect through only perceived psychological help value was not statistically significant (Bootstrap 95% CI [−0.023, 0.008]). Yet the third indirect effect of alcohol consumption on suicide risk through alcohol use as a means to suppress difficult thoughts and feelings and perceived psychological help value in series, with alcohol use to suppress difficult thoughts and feelings modelled as affecting the perceived value of psychological help, which in turn influences suicide risk, was statistically significant (Bootstrap 95% CI [0.001, 0.015]). Pairwise comparisons between specific indirect effects showed that the second indirect effect is not statistically different from the third one (Bootstrap 95% CI [−0.035, 0.003]), but the other pairs of indirect effects differed significantly from each other. Therefore, serial mediation through both alcohol use as a means to suppress difficult thoughts and feelings, and perceived psychological help value is a significant path explaining the link between alcohol consumption and suicide risk in conscripts. Those conscripts who reported drinking more alcohol tended to drink it to suppress difficult thoughts and feelings more often, which in turn was associated with perceived lower value and need of seeking psychological treatment, and this translated into a higher suicide risk.

## 4. Discussion

### 4.1. Comparison of Suicide Risk, Alcohol Consumption, and Attitudes towards Psychological Help between General Population Men, Regular AD Soldiers, and Conscripts

The results of this study of Lithuanian males indicated that military personnel show a significantly lower suicide risk, compared to the general population sample. It is not surprising, because, in general, the military is a highly selected group due to the high physical and mental fitness requirements they need to meet at the enlistment screening before joining the Armed Forces. Although the average suicide risk level did not differ significantly between the regular AD soldiers and conscripts, further analysis showed that the percentage of males at high risk of suicide was particularly low among the regular AD soldiers. The larger percentage of conscripts at high risk of suicide could be in part explained by the stressful challenge that compulsory military service may be generating: leaving home, adapting to strict military rules, daily structure, living conditions, etc. For some conscripts, additional stress can arise from the fact that military service is compulsory rather than voluntary. Although it is known that the majority of conscripts in Lithuania enlist on a voluntary basis, no information regarding the enlistment method was collected in the current study, therefore it was not possible to address the effect of volunteering in the analysis. On the other hand, military service involves mutual dependence and bonding, as well as displays of strong loyalty, which may reduce the risk of suicide [29]. As the effect of bonding and comradeship may build up over time, and conscripts are exposed to the military culture only for a relatively short period of time, they may not fully experience all of the mentioned benefits.

Although a significant, yet small association between alcohol consumption and suicide risk was found in each study group separately and, as previously mentioned, men from the general Lithuanian population showed higher suicide risk, there were no differences in average alcohol consumption across all three study groups: general population men, conscripts and regular AD soldiers. Looking at the percentages of men with risky drinking levels, a slightly lower number was found in the regular AD soldier group, but the difference again was not statistically significant. This is a somewhat unexpected finding, as it is widely recognized that alcohol consumption is one of the major risk factors for suicide, contributing to the emergence and strengthening of suicidal thoughts and the transition from thoughts to suicidal action [30]. This is also demonstrated in studies with military samples. Bryan and colleagues [8] found that among soldiers, alcohol consumption, in general, was associated with a faster transition from a suicidal impulse to action. It is possible that due to very high alcohol consumption levels in Lithuania as a country in general, the association between alcohol consumption level and suicidality is somewhat masked. However, significant differences between study groups were found regarding alcohol consumption as a means to suppress difficult thoughts and feelings. General population Lithuanian men tend to use alcohol as a coping mechanism significantly more often than regular AD soldiers.

The study’s findings suggest that conscripts have significantly more negative attitudes toward professional psychological help in terms of both openness to seeking treatment for emotional problems, and the perceived value of seeking treatment, compared to regular AD soldiers and general population men. The attitudes of the latter groups were very similar and no significant differences were found. These interesting findings might reflect the relationship between mental health stigma and age, as conscripts are predominantly very young adults. Although there is a common misconception that older people have higher levels of stigma as they grew up in times when mental health services were not common, a larger body of work suggests that, in fact, younger people have less positive attitudes than older individuals. For example, a study by Mackenzie and colleagues [31] showed young men having especially high levels of public stigma of male depression and suicide. However, it is important to point out that the significant difference in our study was present when age-adjusted calculations were made. Another possible explanation might be related to common barriers in the military to seeking help. As a systematic review indicated [14], around half of the service members report that they do not need care or it is more advantageous to handle their problems on their own. However, in the current study, regular AD soldiers showed significantly more positive attitudes toward professional psychological help compared to conscripts. Regular AD soldiers throughout their service careers get in contact with military psychologists quite often, e.g., attending training conducted by military psychologists, participating in various prevention programs, or working closely with military psychologists during deployment in international missions. It is possible that because of these contacts, regular AD soldiers might develop more positive attitudes towards the psychology profession in general and thus psychological help as well.

### 4.2. Relationship between Suicide Risk, Alcohol Consumption and Attitudes towards Psychological Help

The results of the current study showed that suicide risk in all study groups was associated more strongly with the frequency of alcohol use as a means to suppress difficult thoughts and feelings than the alcohol consumption level. This was also evident in the results of the multiple regression analyses. In the general population of men and regular AD soldiers, alcohol use as a means to suppress difficult thoughts and feelings remained the only significant predictor of suicide risk, while average alcohol consumption and attitudes towards professional psychological help lost their significance. It seems that when it comes to suicide risk, the reason for using alcohol could be more important than the amounts consumed. Furthermore, alcohol use as a means to suppress difficult thoughts and feelings fully mediated the relationship between alcohol consumption levels and suicide risk in general population men and regular AD soldier samples. A non-direct relationship between alcohol consumption and suicide risk and/or mental health problems, in general, is also shown by other studies in the military field: in the UK military, changes in alcohol use are linked to mental health and life events [32], and both abstainers and heavy drinkers are more likely to have a mental health problem, compared to average drinkers [33]; Bryan and colleagues [8] research in the US military suggested that even low levels of alcohol consumption may be sufficient to promote suicidal behaviour.

Interestingly, only among conscripts, perceived value and need in seeking treatment remained a significant predictor of suicide risk along with alcohol use as a means to suppress difficult thoughts and feelings. Moreover, the serial multiple mediation model confirmed a significant mediating path between alcohol consumption and higher suicide risk through more frequent alcohol use to cope with difficulties and a perceived lower need and value of seeking treatment in the conscript group. Further research is needed to create a richer understanding of more negative attitudes towards professional psychological help-seeking among conscripts. Nonetheless, the results of the current study suggest that there is an opportunity for intervention aimed at the attitudes towards seeking professional psychological help for conscripts. Especially, considering the findings of the current study, that the largest proportion of abstainers were observed among conscripts, yet the average alcohol consumption was similar between all study groups, suggesting that those conscripts who use alcohol, must consume it in larger quantities. The enhancement of mental health literacy (particularly, the perception of value and the need of seeking treatment) among conscripts might lessen the negative effects of alcohol use on suicide risk. However, that might not be the case for regular AD soldiers, nor the general population of Lithuanian men. Although it is possible that such interventions might be valuable for all of those who share less positive attitudes towards professional psychological help, in the case of regular AD soldiers and general population Lithuanian men, interventions aimed at promoting alternative and more adaptive coping techniques than alcohol consumption might be more beneficial in lowering the suicide risk. As alcohol use as a means to suppress difficult thoughts and feelings emerged as a significant mediator of the relationship between alcohol consumption and suicide risk among all study participants regardless of their military or civilian status, it is important to consider possible interventions teaching more adaptive coping techniques that can have long-term benefits for mental health. Examples of such coping techniques could be mindfulness meditation, exercise, and seeking social support. All of these have been shown to be effective alternatives that can reduce symptoms of anxiety and depression, and improve psychological well-being [34,35,36].

### 4.3. Limitations

There are limitations within this study that require acknowledgement. The study relied on cross-sectional data, therefore causality cannot be determined. Moreover, due to limited resources and options available, it was not possible to achieve full representativeness, and only quota-based samples were used in this study. In addition, we relied on self-reported data and unfortunately, no information about the response rate was collected. Despite the large sample size of the current study, it is important to note that a few key variables used in the statistical analysis had outliers in their distribution. Although a large sample allows for a more relaxed view of the assumptions of normality, because it should not have a significant impact on the results of statistical analysis, a certain degree of caution in interpreting the obtained results is necessary. However, the central findings of this study were significant at a more stringent *alpha* level (mostly < 0.001) allowing sufficient confidence in the obtained results. All that considered, this is the largest study on the topic in Lithuania, a country with particularly high suicide rates, and the results of this research are highly valuable.

## 5. Conclusions

This is the first study to investigate the relationship between suicide risk, alcohol consumption, and attitudes towards professional psychological help among Lithuanian military and general male populations. Male soldiers of the Lithuanian Armed Forces, as any other country’s military personnel, are subject to a number of unique occupational stressors, therefore it is reassuring that they experience lower suicide risk in comparison with the Lithuanian general population men. Although alcohol consumption is usually associated with suicide risk, the results of the current study emphasize the significant mediator role of the reason behind alcohol consumption, i.e., to cope with difficulties. This study also showed that there is an opportunity for intervention aimed at the attitudes towards seeking professional psychological help for conscripts, as their attitudes were significantly more negative compared to regular AD soldiers and general population men groups. Furthermore, among conscripts, perceived value in seeking treatment was another significant mediator of the relationship between alcohol use and suicide risk.

## Figures and Tables

**Table 1:** Observed and age-adjusted means, and one-way ANCOVA statistics for study variables between general population men, conscripts, and regular AD soldiers.

Variable	Scale Min.–Max.	General Population Men		Conscripts		Regular AD Soldiers		*F*	*df*	*p*
		Obs. Mean	Adj. Mean	Obs. Mean	Adj. Mean	Obs. Mean	Adj. Mean			
SBQ-R	3–18	5.81	6.16 *a*	4.88	4.45 *b*	3.86	4.03 *b*	57.98	2, 1148	<0.001
AUDIT-C	0–12	3.82	3.79	3.64	3.67	3.31	3.30	2.44	2, 1151	0.087
SUPPRESS	0–4	0.59	0.59 *a*	0.56	0.56	0.42	0.42 *b*	3.37	2, 1157	0.035
VALUE	0–15	8.12	8.50 *a*	7.47	7.02 *b*	8.04	8.20 *a*	13.28	2, 1147	<0.001
OPENNESS	0–15	8.31	8.29 *a*	5.82	5.85 *b*	7.78	7.77 *a*	30.36	2, 1149	<0.001

Note. SBQ-R = suicide risk; AUDIT-C = alcohol consumption; SUPPRESS = alcohol use as a means to suppress difficult thoughts and feelings; VALUE = perceived professional psychological help value; OPENNESS = openness for professional psychological help; ANCOVA = analysis of covariance. Comparisons based upon ANCOVA adjusted means controlling for an age mean of 29.5. Obs. Mean = observed mean; Adj. Mean = age-adjusted mean. Means with different subscripts (i.e., ‘*a*’, and ‘*b*’) differ at the *p* = 0.05 level by Tukey multiple comparisons of the means test.

**Table 2:** Multiple regression analyses with suicide risk (SBQ-R) as a dependent variable.

Sample	Variable	*B*	*SE*	95% CI	*p*
General population men	AUDIT-C	0.01	0.06	[−0.11, 0.13]	0.923
	SUPPRESS	0.67	0.19	[0.30, 1.05]	<0.001
	VALUE	0.05	0.05	[−0.04, 0.14]	0.305
	OPENNESS	0.08	0.05	[−0.01, 0.17]	0.080
	*F*(4, 424) = 6.59, *p* < 0.001, *R*^2^ = 0.06, *R*^2^_adjusted_ = 0.05.				
Conscripts	AUDIT-C	0.004	0.05	[−0.09, 0.09]	0.938
	SUPPRESS	0.67	0.16	[0.36, 0.98]	<0.001
	VALUE	−0.11	0.04	[−0.18, −0.04]	0.003
	OPENNESS	−0.02	0.03	[−0.08, 0.05]	0.602
	*F*(4, 426) = 8.69, *p* < 0.001, *R*^2^ = 0.08, *R*^2^_adjusted_ = 0.07.				
Regular AD soldiers	AUDIT-C	0.04	0.05	[−0.06, 0.14]	0.462
	SUPPRESS	0.40	0.16	[0.08, 0.72]	0.016
	VALUE	−0.01	0.04	[−0.08, 0.06]	0.768
	OPENNESS	0.002	0.03	[−0.06, 0.07]	0.959
	*F*(4, 216) = 2.88, *p* = 0.024, *R*^2^ = 0.05, *R*^2^_adjusted_ = 0.03.				

Note. AUDIT-C = alcohol consumption; SUPPRESS = alcohol use as a means to suppress difficult thoughts and feelings; VALUE = perceived professional psychological help value; OPENNESS = openness for professional psychological help.

**Table 3:** Regression coefficients, standard errors, and model summary information for the mediator model are depicted in Figure 1.

Sample	Antecedent	Consequent							
			*M* (SUPPRESS)				*Y* (SBQ-R)		
			Coeff.	*SE*	*p*		Coeff.	*SE*	*p*
General population men	X (AUDIT-C)	*a*	0.157	0.014	<0.001	*c’*	0.001	0.061	0.993
	M (SUPPRESS)		-	-	-	*b*	0.707	0.187	<0.001
	Constant	*i_M_*	−0.012	0.064	0.852	*i_Y_*	5.390	0.252	<0.001
		*R*^2^ = 0.232 *F*(1, 436) = 131.647, *p* < 0.001					*R*^2^ = 0.041 *F*(2, 435) = 9.299, *p* < 0.001		
Regular AD soldiers	*X* (AUDIT-C)	*a*	0.167	0.018	<0.001	*c’*	0.030	0.049	0.541
	*M* (SUPPRESS)		-	-	-	*b*	0.431	0.153	0.005
	Constant	*i_M_*	−0.141	0.076	0.065	*i_Y_*	3.604	0.178	<0.001
		*R*^2^ = 0.277 *F*(1, 229) = 87.869, *p* < 0.001					*R*^2^ = 0.058 *F*(2, 228) = 6.967, *p* = 0.001		

Note. SBQ-R = suicide risk; AUDIT-C = alcohol consumption; SUPPRESS = alcohol use as a means to suppress difficult thoughts and feelings.

**Table 4:** Regression coefficients, standard errors, and model summary information for the serial multiple mediator model depicted in Figure 2.

Consequent												
Antecedent		*M_1_* (SUPPRESS)				*M_2_* (VALUE)				*Y* (SBQ-R)		
		Coeff.	*SE*	*p*		Coeff.	*SE*	*p*		Coeff.	*SE*	*p*
*X* (AUDIT-C)	*a_1_*	0.123	0.013	< 0.001	*a_2_*	0.057	0.059	0.339	*c’*	0.008	0.05	0.858
*M_1_* (SUPPRESS)		-	-	-	*d_21_*	−0.512	0.205	0.013	*b_1_*	0.669	0.158	< 0.001
*M_2_* (VALUE)		-	-	-		-	-	-	*b_2_*	−0.108	0.037	0.004
Constant	*iM_1_*	0.121	0.060	0.042	*iM_2_*	7.496	0.255	< 0.001	*iY*	5.245	0.338	< 0.001
	*R*^2^ = 0.182 *F*(1, 434) = 96.772, *p* < 0.001				*R*^2^ = 0.014 *F*(2, 433) = 3.128, *p* = 0.045				*R*^2^ = 0.075 *F*(3, 432) = 11.654, *p* < 0.001			

Note. SBQ-R = suicide risk; AUDIT-C = alcohol consumption; SUPPRESS = alcohol use as a means to suppress difficult thoughts and feelings.

## Data Availability

The data for this study are not publicly available. However, corresponding author can be contacted for any additional information around data collection and analyses.

## Supplementary Materials

The following supporting information can be downloaded at: https://www.mdpi.com/article/10.3390/ijerph20043457/s1, Table S1: Descriptive statistics of study variables; Figure S1: Box plots of study variables.

## Appendix A

**Table A1:** Sociodemographic characteristics of all three study samples.

	General Population Men (*n* = 445)	Conscripts (*n* = 490)	Regular AD Soldiers (*n* = 260)
Age group			
18–29	35%	100%	49%
30–39	24%	-	24%
40–49	19%	-	22%
50–59	22%	-	5%
Place of residence			
Major City	45%	51%	51%
Town	25%	19%	34%
Rural location	30%	30%	15%
Marital status			
Married	47%	2%	49%
Never married	46%	95%	43%
Divorced	5%	3%	7%
Widower	1%	-	1%

## Appendix B

**Table A2:** Correlations for study variables in the general population men sample.

	1.	2.	3.	4.	5.
1. SBQ-R	-				
2. AUDIT-C	0.09 *	-			
3. SUPPRESS	0.20 ***	0.48 ***	-		
4. VALUE	0.12 **	−0.03	0.02	-	
5. OPENNESS	0.13 *	−0.01	0.04	0.54 ***	-

Note. SBQ-R = suicide risk; AUDIT-C = alcohol consumption; SUPPRESS = alcohol use as a means to suppress difficult thoughts and feelings; VALUE = perceived professional psychological help value; OPENNESS = openness for professional psychological help; * *p* < 0.05, ** *p* < 0.01, *** *p* < 0.001.

**Table A3:** Correlations for study variables in the conscript sample.

	1.	2.	3.	4.	5.
1. SBQ-R	-				
2. AUDIT-C	0.10 *	-			
3. SUPPRESS	0.25 ***	0.41 ***	-		
4. VALUE	−0.16 ***	−0.01	−0.12 *	-	
5. OPENNESS	−0.02	−0.02	0.07	0.04	-

Note. SBQ-R = suicide risk; AUDIT-C = alcohol consumption; SUPPRESS = alcohol use as a means to suppress difficult thoughts and feelings; VALUE = perceived professional psychological help value; OPENNESS = openness for professional psychological help; * *p* < 0.05, *** *p* < 0.001.

**Table A4:** Correlations for study variables in the regular AD soldier sample.

	1.	2.	3.	4.	5.
1. SBQ-R	-				
2. AUDIT-C	0.17 *	-			
3. SUPPRESS	0.23 ***	0.53 ***	-		
4. VALUE	-0.05	-0.10	-0.04	-	
5. OPENNESS	-0.04	-0.20 *	-0.10	0.33 ***	-

Note. SBQ-R = suicide risk; AUDIT-C = alcohol consumption; SUPPRESS = alcohol use as a means to suppress difficult thoughts and feelings; VALUE = perceived professional psychological help value; OPENNESS = openness for professional psychological help; * *p* < 0.05, *** *p* < 0.001.
