# Supplementary material for: Suicide Risk, Alcohol Consumption and Attitudes towards Psychological Help-Seeking among Lithuanian General Population Men, Conscripts and Regular Active Duty Soldiers
Source: Int J Environ Res Public Health. 2023 Feb 16;20(4):3457. doi: 10.3390/ijerph20043457 (PMC9961175; doi:10.3390/ijerph20043457)
Supplement: Supplementary file 1 [file ijerph-20-03457-s001.zip › ijerph-2178956-supplementary.pdf]

Supplementary Materials

Table S1. Descriptive statistics of study variables.

Variable	Min.-max.	Mean	SD	Skewness	Kurtosis
SBQ-R	3-18	5.02	2.78	1.88*	3.86*
AUDIT-C	0-12	3.63	2.83	0.5	-0.34
SUPPRESS	0-4	0.54	0.86	1.72*	2.66*
VALUE	0-15	7.83	3.55	0	-0.52
OPENNESS	0-15	7.17	3.98	-0.15	-0.84

Note. SBQ-R = suicide risk; AUDIT-C = alcohol consumption; SUPPRESS = alcohol use as a means to suppress difficult thoughts and feelings; VALUE = perceived professional psychological help value; OPENNESS = openness for professional psychological help; * Skewness and kurtosis higher than would be expected in a normally distributed measure.

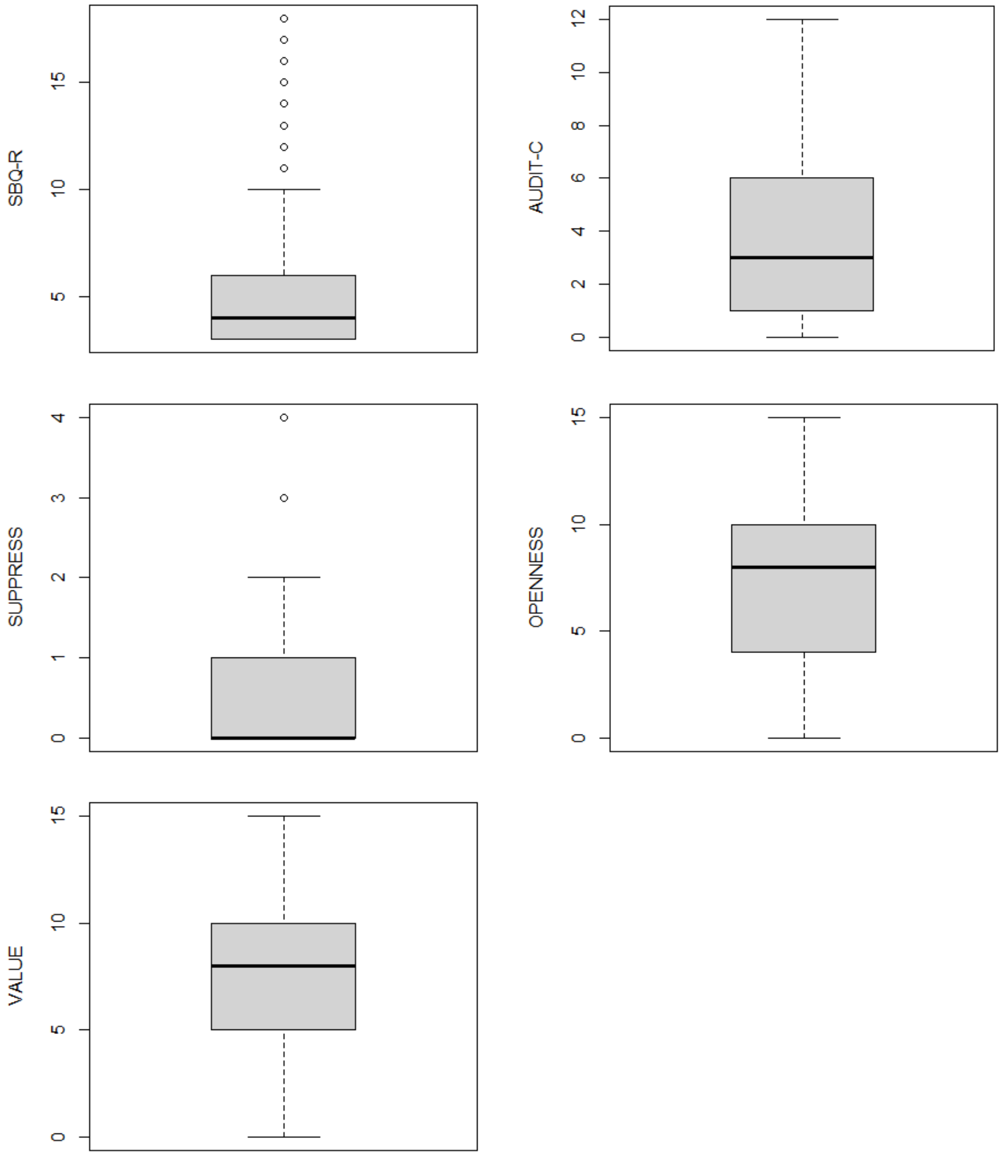

Figure S1. Box plots of study variables.
